# An Account of the Method of Making a Wine, Called by the Tartars Koumiss; with Observations on Its Use in Medicine

**Published:** 1789

**Authors:** John Grieve

**Affiliations:** late Physician to the Russian Army


VIII. *An Account of the Method of making a Wine, called by the Tartars Koumiſs; with Obſervations on its Uſe in Medicine. By* John Grieve, *M. D. F. R. S. Edin. and late Phyſician to the Ruſſian Army.* Vide *Tranſactions of the Royal Society of Edinburgh*, Vol. I. 4to. Edinburgh, 1788.

THE vinous liquor here deſcribed is procured by fermentation from mare's milk; but although it has for ſome ages been employed by ſeveral tribes of Tartars, Dr. Grieve obſerves that even in Ruſſia it was with difficulty he could learn the particulars of the method of preparing it.

In

In books he has met with little ſatisfactory information concerning it. All the writers who mention it agree, it ſeems, that a vinous liquor from mare's milk is uſed by ſome of the Tartar nations under the name of *Koumiſs*; but none of them deſcribe the mode of preparing it, or the purpoſes in œconomy or phyſic to which it is applicable.

Marcus Paulus Venetus, in a work publiſhed ſo long ago as the thirteenth century *, ſpeaks of it as the common beverage of the Tartars, but makes no mention of the method of preparing it.

Strahlenberg, in his deſcription of the Ruſſian empire †, relates ſome circumſtances of the preparation; but his method, our author obſerves, if followed, could not be attended with ſucceſs: for he mentions that the Kalmucks take off the thick ſubſtance, which, in conſequence of ſouring, riſes to the top of the milk, and employ this in their food, while they uſe the remaining liquor either for drink or diſtillation. This, Dr. Grieve farther remarks, is not only contrary to the uſage of that people, when

* De Region. Oriental. lib. 1, cap. 57.

† Beſchreibung des Ruſſiſchen Reichs, p. 319.

they wiſh to obtain a fermented liquor of any ſtrength, but experience, he adds, proves that no perfect fermentation can be produced unleſs all the parts of the milk be left united in their natural proportion.

Of Gmelin, our author remarks, that, in the account of his tour through Siberia *, he attends more to the Tartar method of diſtilling a ſpirit from the wine of milk than to the fermenting proceſs by which that wine is procured.

The account given of Koumiſs by Dr. Pallas, in the Hiſtory of his Travels through ſome of the Provinces of the Ruſſian Empire †, is allowed by Dr. Grieve to be as circumſtantial as could well be expected from a traveller whoſe object was natural hiſtory in general; but he obſerves at the ſame time that the principles on which the fermentation depends, as well as the mode of conducting the proceſs, are not ſufficiently explained in that work.

After pointing out ſome erroneous aſſertions relative to this ſubject, which occur in the wri-

* Reiſſe durch Siberien, T. I. p. 273.

† Phyſ. Reiſe durch einig. Provintz. des Ruſſiſch. Reichs, T. I. p. 316.

tings tings of Newman *, Voltelen †, and Macquer ‡, our author proceeds to defcribe a method of preparing Koumifs which is common among the Bafchkir Tartars, and which he has adopted in his own practice with fuccefs. This procefs was communicated to him by a Ruffian Nobleman who refided for fome time among thefe people for the purpofe of drinking this liquor, and is as follows:

"Take of frefh mare's milk, of one day, " any quantity; add to it a fixth part of wa- " ter, and pour the mixture into a wooden vef- " fel; ufe then, as a ferment, an eighth part " of the foureft cow's milk that can be got; " but, at any future preparation, a fmall por- " tion of old Koumifs will better anfwer the " purpofe of fouring; cover the veffel with a " thick cloth, and fet it in a place of moderate " warmth; leave it at reft twenty-four hours, " at the end of which time the milk will have " become four, and a thick fubftance will be " gathered on the top; then with a ftick, made

* Chem. Exp. T. I. Part 2, p. 18.

† Obf. de lacte humano cum afinino et ovillo comparato, p. 54.

‡ Dict. of Chemiftry, p. 432.

" at the lower end in the manner of a churn-

" ſtaff, beat it till the thick ſubſtance above

" mentioned be blended intimately with the

" ſubjacent fluid. In this ſituation leave it

" again at reſt for twenty-four hours more;

" after which pour it into a higher and nar-

" rower veſſel, reſembling a churn, where the

" agitation muſt be repeated, as before, till the

" liquor appears to be perfectly homogeneous;

" and in this ſtate it is called *Koumiſs*; of

" which the taſte ought to be a pleaſant mixture

" of ſweet and ſour. Agitation muſt be em-

" ployed every time before it be uſed."

To this detail of the proceſs our author has added ſome other points of information he has been able to collect relative to this ſubject. Thus by one Tartar, who lived to the ſouth eaſt of Orenbourg, he has been told, that, to prevent changing the veſſel, the milk may be put at once into a pretty high and narrow veſſel, and that, in order to accelerate the fermentation, ſome warm milk may be added to it, and, if neceſſary, more ſouring. From another Tartar he learned that the proceſs may be much ſhortened by heating the milk before the ſouring is added to it, and as ſoon as the parts begin to ſeparate, and a thick ſubſtance to riſe to the top, by agitating it every hour, or oftener. In this way, we are told, he made ſome in the author's preſence in the ſpace of twelve hours. From this perſon Dr. Grieve learned alſo, that, among ſome Tartars, it is common to prepare it in one day during ſummer, and that with only two or three agitations; but that in winter, when, from a deficiency of mare's milk, they are obliged to add a great proportion of that of cows, more agitation and more time are neceſſary. He was likewiſe informed, that, when well ſecured in cloſe veſſels, and kept in a cold place, it may be preſerved for three months, or even more, without any injury to its qualities.

The ſame Tartar ſaid farther, that, inſtead of employing ſour milk to produce the acid fermentation, the ſame effect might be obtained from a ſour paſte of rye flour, from the rennet of a lamb's ſtomach, or, what is more common, from a portion of old Koumiſs; and that, in ſome places, they ſaved much time by adding the new milk to a quantity of that already fermented, on being mixed with which it very ſoon undergoes the vinous change.

From this Tartar our author purchaſed one of the leathern bags which are uſed by the Kalmucks for the preparation and carriage of their

Koumifs. This bag was made of a horfe's hide undreffed, and, by having been fmoked, had acquired a great degree of hardnefs. Its fhape was conical, but at the fame time fomewhat triangular, from being compofed of three different pieces fet in a circular bafe of the fame hide. It had a dirty appearance, and a very difagreeable fmell. The Tartar, on being afked the reafon of this, faid, " the remains of " the old Koumifs were left, in order to fupply " a ferment to the new milk."

From all the preceding accounts it appears that three things are effential to the vinous fermentation of milk. Thefe are *heat*, *fouring*, and *agitation*. Our author remarks, that heat is neceffary to every fpecies of fermentation, and fouring perhaps not lefs fo, but that the chief art of fermenting milk confifts in agitation. In fermenting vegetable juices and infufions, nature, he obferves, has no need of the affiftance of art; the inteftine motion that accompanies the fermentation being fufficient to produce the degree of agitation which feems neceffary to keep the parts of the fluid in mutual contact, or to fit them for mutual action; while milk, on the contrary, is no fooner foured than a feparation of its parts takes place; the cream rifes to the top, while the cheefe either falls to the bottom, or is fufpended in the whey. When thefe parts are brought, however, into clofe contact with one another, by agitation, and this repeated at proper intervals, a vinous liquor is produced, of the medical virtues of which Dr. Grieve next proceeds to treat.

From the time he firft heard of Koumifs he conceived, it feems, an opinion of its importance in the cure of certain difeafes. He judged that a preparation of milk, which could not be curdled by the juices of the ftomach, while, at the fame time, it poffeffed all its nutritive qualities, with the fuperaddition of a fermented fpirit, might be of effential fervice in thofe diforders in which the body is defective either in nourifhment or ftrength.

The cafe of the above-mentioned Nobleman, who communicated to him the firft procefs, gave him an opportunity of trying how far his conjectures were well founded. The patient was in a ftate that feemed ftrongly to indicate the ufe of fuch a medicine as Koumifs, and our author accordingly advifed him to it.

The patient was twenty-fix years of age, and his complaints had arifen from the injudicious treatment of a confirmed lues venerea, for which he had undergone three ſucceſſive ſalivations by mercury. He was much emaciated; his face was of a livid yellow colour; his eyes were ſunk; he felt a ſevere pain in his breaſt, accompanied with a conſiderable cough and mucous expectoration; his appetite and digeſtion were greatly impaired; and he had frequent tremblings and faintings. In a word, we are told that his whole appearance was conſumptive, that he was beginning to feel the ſymptoms of hectic fever, and was ſo weak, that he required aſſiſtance to get into the carriage in which he was to be conveyed into Tartary.

After drinking Koumiſs ſix weeks only he returned perfectly free from all the above ſymptoms, and was become ſo plump and freſh coloured, that, at firſt ſight, it was with difficulty his friends could recogniſe him.

It ſeems that ſoon after he began to take the Koumiſs (which ſerved him both for food and drink) he ceaſed to be diſturbed in his ſleep; his nervous and dyſpeptic ſymptoms left him, and he became cheerful; and though he uſed it to the quantity of a gallon and a half, and ſometimes even more, in the twenty-four hours, yet he always drank it with pleaſure and without intoxication. His body, during its uſe, was regularly regularly open, and the quantity of his urine much increafed.

Befides this inftance of the falutary effects of Koumifs, three others are particularly defcribed by the author. The fubject of the firft of thefe was a lady who had long laboured under a train of nervous fymptoms, by which fhe was reduced to a ftate of extreme weaknefs, and who was reftored to health in about a month by the ufe of Koumifs, which was fent to her in cafks, well fecured. The fecond patient was a young gentleman who had all the fymptoms of incipient phthifis, for which a diet chiefly of milk had been tried without any figns of recovery. This patient drank Koumifs for about two months only, and that at an unfavourable feafon, but the confequence was that all his complaints difappeared, and his flefh and ftrength returned. In the third inftance the patient laboured under an abfcefs in the left fide, accompanied with all the appearances of incipient hectic. In this cafe a cure was obtained in about fix weeks by the ufe of Koumifs, proper chirurgical dreffings being at the fame time applied to the wound.

There were fome other cafes in which our author employed Koumifs with the fame good effects as in thofe we have mentioned; but of thefe, these, as being less important, he has not thought it neceſſary to give any particular account. He informs us, however, that all thoſe who drank it agreed in ſaying, that, "during its uſe, they "had little appetite for food; that they drank "it in very large quantities, not only without "diſguſt, but with pleaſure; that it rendered "their veins turgid, without producing lan-"guor; that, on the contrary, they ſoon ac-"quired from it an uncommon degree of "ſprightlineſs and vivacity; and that even in "caſes of ſome exceſs it was not followed by "indigeſtion, head-ach, or any of the ſymp-"toms which uſually attend the abuſe of other "fermented liquors." To this, he obſerves, may be added, that the Baſchkir Tartars, who, towards the end of winter, are much emaciated, no ſooner return in ſummer to the uſe of Koumiſs than they become ſtrong and fat *.

* Dr. Grieve, in a note to this part of his paper, refers to a deſcription lately publiſhed of the Ruſſian Empire, the author of which, ſpeaking of Koumiſs, ſays, "Elle eſt fort "nouriſſante, et peut tenir lieu de tout autre aliment. Les "Baſchkirs s'en trouvent tres bien; elle les rend bienportans "et gais; elle leur donne de l'embonpoint, et de bonnes "couleurs." — *Deſcript. de toutes les Nat. de l'Emp. Ruſſ.* T. II. p. 118.

From

From all thefe circumftances our author thinks it may be inferred that this wine of mare's milk may be applied to many medicinal purpofes. From its mild acid, its vinous fpirit, and its oily and mucilaginous qualities, may it not, he afks, be confidered as a cooling antifeptic, as an ufeful ftimulant and tonic, or as a valuable article of nourifhment? Thefe confiderations lead him (but with great diffidence, and in the way of query only) to fuggeft a trial of it in acute difeafes attended with marks of weaknefs and putridity; in cafes of exceffive irritability; in dyfpepfia; in phthifis; and in all cafes of fuppuration or ulcer in which the body is threatened with hectic fever.

From the fcarcity of mare's milk in this country. inquiries will naturally be made whether other fpecies of milk admit of a fimilar vinous fermentation, and what proportion of fpirit they contain. Thefe, it feems, have never been the object of our author's attention; but he gives us the fubftance of what he has been able to learn from others refpecting that which is the moft common, the milk of cows.

Dr. Pallas, in the work already quoted *, fays,

* Phyf. Reife durch verfchied. Provintz. des Ruffifch. Reichs, T. I. p. 316.

that that the Tartars, when mare's milk fails them, prepare a wine from cow's milk; but that they always prefer Koumifs when it can be got, as it is more agreeable, and contains a greater quantity of fpirit, of which it yields, on diftillation, one-third part of its whole quantity; while from the wine of cow's milk, or *airen*, as it is called, only two-ninth parts of fpirit are obtained.

This account our author finds confirmed by Oferetfkowfky, who accompanied Lepechin and other Academicians in their travels through Siberia and Tartary, and who publifhed in 1778, at Strafburgh, a Differtation* on the ardent Spirit to be obtained from Cow's Milk. From his experiments it appears that cow's milk may be fermented with, or even without, fouring, provided fufficient time and agitation be employed; that no fpirit could be produced from any one of its conftituent parts taken feparately, nor from any two of them, unlefs inafmuch as they were mixed with fome part of the third; that the milk, with all its parts in their natural proportion, was the moft productive of it; that the clofer it was kept, or, which is the fame thing, the more difficultly the fixed air is allowed

* Specim. inaug. de Spir. Ard. ex lact. Bub.

to eſcape during the fermentation, (care being taken, however, that we do not endanger the burſting of the veſſel) the more ſpirit is obtained. The ſame writer farther obſerves, that it had a ſourer ſmell before than after agitation; that the quantity of ſpirit was increaſed by allowing the fermented liquor to repoſe for ſome time before diſtillation; that from ſix pints of milk, fermented in a cloſe veſſel, and thus ſet to repoſe, he obtained three ounces of ardent ſpirit; but that from the ſame quantity of the ſame milk, fermented in an open veſſel, he could ſcarcely obtain one ounce.

